# Metropolitan Hospital Sunday Fund

**Published:** 1908-12-05

**Authors:** 


### METROPOLITAN HOSPITAL SUNDAY FUND.

A Meeting of the Council of the Metropolitan Hospital Sunday Fund was held at the Mansion House on Monday, November 30, the Lord Mayor, Sir George Wyatt Truscott, President and Treasurer of the Fund, in the chair.

The Bishop of London moved the adoption of the Report of the Council for the year ended October 31, 1908, which states that the year's collection, the thirty-sixth, resulted, under the Presidency and Treasurership of the Right Hon. Sir John Charles Bell, Bart., Lord Mayor, in a total of £80,181 15s. 9d., the collections in the various places of worship on Hospital Sunday, June 21, 1908, resulting in a sum of £40,239, being £2,547 less than in 1907, which is attributable to various causes, the chief of which being believed to have been the "Pan-Anglican Congress" collection. The Metropolitan Cathedral of St. Paul's heads the list with £4,510; St. Michael's, Chester Square, the late Rev. Canon Fleming, comes second with £1,187; and Christ Church, Lancaster Gate, Rev. Preb. Gurdon, third with £1,003.

The sum of £35,000 was received from the executors of the late Mr. George Herring; and a further instalment of £2,000 from the executors of the late Mr. Herbert Lloyd, on account of the legacy of £10,000 bequeathed by him to the Fund in 1901. Mr. William Herring gave a donation of £1,000; Sir Savile Crossley again divided his contribution of £1,000 between the Sunday Fund and the King's Fund, and "Delta" sent his twenty-ninth donation of £200.

The Report further states that the Council views with apprehension the extensions which are in contemplation by certain hospitals and institutions whose reliable income at the present time by no means meets the expenditure, and where a considerable number of beds are empty for want of funds. The Council is of opinion that in the present state of hospital finances extensions and additions should be discouraged by this Fund, except in cases where the hospital can show that it has the means to provide and maintain them. The adoption of the Report was seconded by the Rev. Hardy Harwood and carried *nem. con.*

The Archdeacon of London, on the ground that the Fund had been placed in a new position by the Herring Bequest of £600,000, and that continuity of principle and of action are now more than ever desirable, moved the following resolution, which was also carried without dissent:

"That the Lord Mayor for the time being continue to hold the office of President and Treasurer of the Fund, and that a Deputy-Chairman be appointed, such office to be annual like those of the Chairmen of the Committees, the late Lord Mayor, Sir John C. Bell, Bart., being asked to become the first holder of the office."

On the report of the General Purposes Committee, Wednesday, December 16, was fixed for the Annual Meeting of Constituents, and June 13 for Hospital Sunday, 1909; and this concluded the business of the meeting.

